# Genotoxicity of lapachol evaluated by wing spot test of *Drosophila melanogaster*

**DOI:** 10.1590/S1415-47572010005000070

**Published:** 2010-09-01

**Authors:** Wender Ferreira Costa, Alaide Braga de Oliveira, Júlio César Nepomuceno

**Affiliations:** 1 Instituto de Genética e Bioquímica, Universidade Federal de Uberlândia, Uberlândia, MGBrazil; 2 Departamento de Produtos Farmacêuticos, Faculdade de Farmácia, Universidade Federal de Minas Gerais, Belo Horizonte, MGBrazil; 3 Laboratório de Citogenética e Mutagênese, Centro Universitário de Patos de Minas, Patos de Minas, MGBrazil

**Keywords:** *Drosophila melanogaster*, SMART, lapachol, doxorubicin, cytochrome P450

## Abstract

This study investigated the genotoxicity of Lapachol (LAP) evaluated by wing spot test of *Drosophila melanogaster* in the descendants from standard (ST) and high bioactivation (HB) crosses. This assay detects the loss of heterozygosity of marker genes expressed phenotypically on the fly's wings. *Drosophila* has extensive genetic homology to mammals, which makes it a suitable model organism for genotoxic investigations. Three-day-old larvae from ST crosses (females *flr*^*3*^*/TM3, Bd*^*s*^ x males *mwh/mwh*), with basal levels of the cytochrome P450 and larvae of high metabolic bioactivity capacity (HB cross) (females *ORR; flr*^*3*^*/TM3, Bd*^*s*^ x males *mwh/mwh*), were used. The results showed that LAP is a promutagen, exhibiting genotoxic activity in larvae from the HB cross. In other words, an increase in the frequency of spots is exclusive of individuals with a high level of the cytochrome P450. The results also indicate that recombinogenicity is the main genotoxic event induced by LAP.

Approximately two-thirds of the biological diversity worldwide occurs in tropical zones, mainly in developing countries, especially in Brazil with several different biomes (Vieira, 1999). The *Cerrado*, the second largest ecological domain, covers approximately 25% of the total surface area, with a continuous herbaceous stratum joined to an arboreal one, with variable density of woody species, and Around 220 of those species reportedly used in traditional medicine (Vieira and Martins, 1998).

Lapachol (LAP), 4-hydroxy-3-(3-methylbut-2-enyl)naphthalene-1,2-dione, is a naphthoquinone found in many vegetable species of the *Bignoniaceae* family, specifically those of the genus *Tabebuia* (*Tabebuia aurea*, *Tabebuia impetiginosa*, *Tabebuia ochracea*) from the *Cerrado*. It is endowed with antimicrobial qualities active in combating bacterial, fungal and virus infections, as well as, and more importantly, cancer (Fonseca *et al.*, 2004; Castellanos *et al.*, 2009). This quinone showed significant *in vivo* anti-tumor activity in several early mouse models (Rao *et al.*,1968), since then progressing to clinical trials by the National Cancer Institute (NCI) in the 1970's. However, in 1974, the NCI concluded that the high concentrations required for efficient chemotherapy in human cancer treatment, unfortunately also gave rise to extremely toxic side-effects, thereby justifying its rejection (Suffness and Douros, 1980; Castellanos *et al.*, 2009). Notwithstanding, recent results have shown that lapachol, isolapachol and its acetylderivative are significantly active against *Biomphalaria glabrata*, the intermediate host of *Schistosoma mansoni* (Santos *et al.*, 2000; Lima *et al.*, 2002). According to Lima *et al.* (2004), antileishmanial activity was found to be efficient against certain viral strains including herpes virus Types I and II (Silva *et al.*, 2002). LAP proved to be a vitamin K-antagonist antigen, thus possibly targeting vitamin K-dependent reactions (Dinnen and Ebisuzaki, 1997), besides also being bio-activated by P450 reductase to reactive species which promote DNA scission, through redox cycling with generation of free radicals (Kumagai *et al.*, 1997). The enzyme responsible for bioactivating lapachol, thereby leading to the generation of ROS capable of causing DNA damage, was unknown. An immunoinhibition study with antibodies against cytochrome P450 reductase (P450R), revealed that P450R was a predominant enzyme in catalyzing the one-electron reduction of lapachol (Kumagai *et al.*, 1997).

Generation of reactive oxygen species, superoxide anion radical and hydroxyl radical during the metabolism of LAP by P450 reductase, was confirmed by acetylated cytochrome reduction assay in the absence and presence of Cu, Zn-SOD (Superoxide Dismutase), and electron spin resonance (ESR) studies (Kumagai *et al.*, 1997).

Certain synthetic derivatives of lapachol, such as mono-(arylimines)-o quinones derived from ß-lapachone, also inhibit the activity of the enzyme topoisomerases (Esteves-Souza *et al.*, 2007). DNA supercoiling is a precisely regulated process that influences DNA replication, transcription and packaging. The DNA topoisomerases are enzymes that modulate the topological state of DNA.

The wing somatic mutation and recombination test (SMART), when using *Drosophila melanogaster,* is capable of detecting a vast range of genetic abnormalities, such as mutations, deletions and somatic recombinations (Graf *et al.*, 1984; Würgler *et al.*, 1984). During the embryonic development of *D. melanogaster*, imaginal disc-cell groups proliferate mitotically during larvae growth, until reaching the point of differentiating during metamorphosis of body structures of the adult insect. If genetic alteration occurs in any one of the imaginal disc cells, these changes will be present in all the following cells, subsequently forming a mutant cell clone. This being the case, mutant cells will be detected as a spot of mutant trichome on adult insects' wings (Guzmán-Rincón and Graf, 1995).

The drug doxorubicin (DXR) is capable of creating a variety of free radicals in cells, this capability being considered critical in its anti-tumoral effect (Keizer *et al.*, 1990). The production of free radicals acts directly on the nucleus, thereby generating unfavorable conditions for cell division. This cytotoxic mechanism appears to be the principal antitumoral effect of DXR (Keizer *et al.*, 1990). Doxorubicin is also the genotoxic agent that inhibits topoisomerase II activity, with the consequential accumulation of DNA strand breaks which, if not repaired by the cell, can provoke mutations and chromosomal aberrations (Islaih *et al.*, 2005). In *D. melanogaster*, DXR, analyzed through SMART testing, was classified as a strong mutagen capable of inducing all types of spots (Frei *et al.*, 1985).

Hence, the objective of the present study was to evaluate the genotoxic effects of LAP by applying the *Drosophila melanogaster* wing spot test. The influence of differences in the level of cytochrome P450 on LAP genotoxic activity were evaluated by way of standard (ST) and high-bioactivation (HB) crosses of Drosophila. An HB cross is characterized by an increased cytochrome P450-dependent bioactivation capacity for promutagens when compared with an ST.

Each ampoule of the DXR commercially known as Adriblastina^®^ RD (CAS 23214-92-8) (lot nº G0421), manufactured by Pharmacia & Upjohn S.p.A., Milan, Italy, and imported and distributed by Pharmacia of Brazil, Ltd., contains chlorohydrate of doxorubicin (10 mg), methylparabene (1 mg) and lactose (50 mg), with Registry Number 1.2389.0046 in the Ministry of Health.

Lapachol (CAS 84-79-7) was provided by Dr. A. B. Oliveira (Federal University of Minas Gerais, Belo Horizonte, Minas Gerais, Brazil). The molecular structures of the test drugs are depicted in Figure 1. Solutions of these compounds were prepared with ethanol 5% just before use.

Three mutant strains of *Drosophila melanogaster* (*ORR*, *flr*^*3*^ and *mwh*), with the genetic markers of *multiple wing hairs* (*mwh*, 3-0.3) and *flare-3 (flr*^*3*^, 3-38.8), were used.

Female virgins *flr*^*3*^*/In(3 LR)TM3*, *ri pp sep I(3)89Aa bx*^*34e*^*and Bd*^*s*^ were crossed with *mwh/mwh* males to produce the ST cross (Graf *et al.*, 1989). The high metabolic bioactivation cross, with high constitutive levels of cytochrome P450 was the result of crossing the female virgins *ORR/ORR*; *flr*^*3*^*/In(3 LR)TM3*, *ri p*^*p*^*sep I(3)89Aa bx*^*34e*^*and Bd*^*s*^ with *mwh/mwh* males (Graf and van Schaik 1992).

The resultant larvae of both genotypes were simultaneously treated with LAP, to facilitate future contact with the chemical agents to be tested. Larval descendents were collected over an 8 h period in culture jars containing a solid agar base (3% of agar in water), with the addition of a layer of live baker's yeast (*Sachcaromyces cerevisiae*) and sugar. After three days (72 + 8 h), the larvae were washed out with tap water through a fine-mesh stainless steel strainer.

Larvae from both crosses were transferred to glass tubes, 2.5 cm in diameter and 8.0 cm high, each containing 1.5 g of instant mashed potatoes (HIKARI, Lot nº L3068DD, São Paulo, Brazil) and 5.0 mL of LAP (20, 40 and 60 μg/mL). The concentrations used in this experiment were based on studies of the lethal dose of lapachol in *Aedes aegypti* larvae (Rodrigues *et al.*, 2005). DXR (0.125 mg/mL) constituted the positive control, whereas ethanol 5% was used as the negative. As some compounds were photosensitive, all the tubes were wrapped in aluminum foil. Both control and treated larvae fed on the mashed potatoes until pupation (48 h).

**Figure 1:**
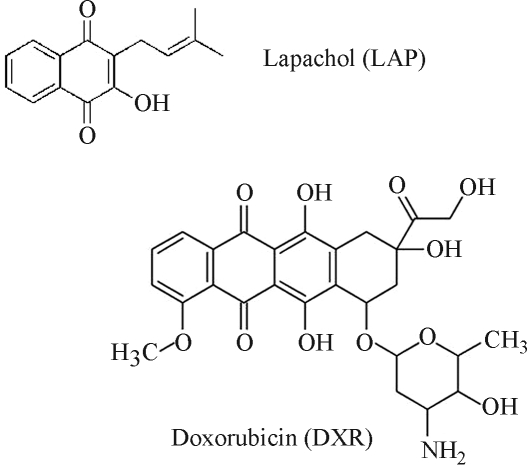
Chemical structures of LAP and DXR.

Each cross produce two types of progeny, that is, marker-heterozygous (MH) (*mwh flr*^*+*^*/mwh*^*+*^*flr*^*3*^) and balancer-heterozygous (BH) (*mwh flr*^*+*^*/mwh*^*+*^*TM3*, *Bd*^*s*^) flies, The dominant *Bd*^*s*^ marker allows the wings of these two genotypes to be distinguished.

The agents tested (LAP and DXR) were prepared in ethanol 5% when the larvae were treated. All experiments were performed at a temperature of (25 ± 2 °C) and at a humidity of 65%. After hatching, the individual adults that emerged were transferred into a recipient containing 70% ethanol, and the wings were mounted on slides with Faure's solution, and analyzed under a compound microscope at 400x magnification (Graf *et al.*, 1984). Frequency and the size of single and twin spots were recorded.

The statistical analysis of the experiment to verify possible genotoxic action of LAP was carried out using a test described by Frei and Würgler (1988), which makes four different diagnoses: positive, weakly positive, negative, or inconclusive. The frequency of each type of mutant clone per fly of a treated series was compared pairwise (*i.e.*, control *vs.* LAP) using the conditional binomial test of Kastenbaum and Bowman (1970). For the final statistical analysis of all positive outcomes, the nonparametric Mann-Whitney *U*-test with significance levels α = β = 0*.*05 was used to exclude false positives (Frei and Würgler 1995).

All the compounds were tested in two different experiments. The data were pooled after verifying that the two independent experiments were in agreement with good reproducibility. Table 1 shows the frequency of mutant spots observed in the marker-heterozygous and balancer-heterozygous descendants of ST cross and HB cross. Statistically, significant elevation was not observed (p > 0.05) on the frequencies of the spots induced by Lapachol (LAP) in the ST cross, in the three treated doses, when compared to the negative control group, in all the categories of spots. On the contrary, there was a positive response in HB descendents, with increased frequency of small single and total spots (for 20, 40 and 60 μg/mL LAP). DXR produced a positive response in both ST and HB descendants, thereby confirming its genotoxicity.

Sousa *et al.* (2009) showed that a commercial preparation of the powdered bark and stem of *Tabebuia impetiginosa* , although toxic, did not induce somatic mutation and recombination in *D. melanogaster* from ST and HB crossbreeding. The absence of genotoxicity, in this case, could be due to the low concentration of lapachol in exposed larvae. However, these authors indicated that powdered bark and stem of *T. impetiginosa* possess a considerable potentiating effect on DXR genotoxicity.

The analysis of flies with genotype *mwh/TM3* was carried out for the purpose of calculating the portion of recombinogenic and mutagenic events. It is possible to separate mutational events from recombinational events, because the recombinational events are eliminated in flies with this genotype. A comparison of clone-induction frequencies obtained for DXR in both genotypes indicated that in ST flies, 12% of mutant clones produced by DXR were due to mutation and 88% to recombination. Furthermore, the very same analysis showed that in HB flies, 21% of spots induced by DXR were due to mutation, and 79% to recombination. The strong recombinogenic activity of DXR in somatic cells of *D. melanogaster* was earlier reported by Lehmann *et al.* (2003), Costa and Nepomuceno (2006) and Fragiorge *et al.* (2007). Our results indicated that recombinogenicity is the major genotoxic effect of LAP 20 μg/mL (approximately 67% through recombination), LAP 40 μg/mL (approximately 65.5% recombination) and LAP 60 μg/mL (approximately 70% recombination). There are no published articles on LAP genotoxicity, and the mutagenicity of this chemical was only studied on the Ames test (Krishnan and Bastow, 2000). On the other hand, mitotic recombinogenic activity had neither been demonstrated nor otherwise quantified. This recombinogenic activity is demonstrated in this study and also found in DXR (another quinone), which again shows similarities in the effects of these drugs.

Numerous quinones play vital roles in the biochemistry of living cells, and exert relevant biological activities. The cytostatic and antimicrobial activities of these quinones emerge by virtue of their ability to act as potential inhibitors of electron transport, as uncouplers of oxidative phosphorylation, as DNA intercalating agents, as bioreductive alkylating agents, and as producers of reactive oxygen radicals by redox cycling under aerobic conditions (Lima *et al.*, 2002).

The main LAP toxicity attribute is its capacity to induce oxidative stress (Silva *et al.*, 2003). It has been demonstrated (Kumagai *et al.*, 1997; Kumagai and Shimojo, 2002) that oxidative stress induced by LAP occurs as a response to P450 reductase enzyme activities, thereby causing changes in the DNA. LAP itself has no direct effect on DNA, although, as can be observed, this is not so when it interacts with cytochrome P450. Similar results were obtained in our study that showed an increase in the frequencies of the mutant spots induced by LAP in descendants from HB cross. DNA changes require bioactivation as processed by cytochrome P450 reductase (CPR), a diflavin enzyme (Kumagai *et al.*, 1997). Shiah *et al.* (1999) were able to demonstrate that beta-lapachone (a semi- synthetic derivate of LAP) is capable of increasing the levels of H_2_O_2_ and O_2_^•-^ (oxidative stress) in leukemia cells of HL-60, thus inducing apoptosis in the later stages. They also showed that induced apoptosis can be related or linked to increased cell H_2_O_2_ levels when activated by NAD (P)H/ quinone oxidoreductase.

Quinones are used as a chemotherapy agent since they act much like LAP. Doxorubicin (DXR) is capable of producing a series of free radicals in the cell (Keizer *et al.*, 1990). These free radicals produced by DXR act directly on the nucleus, generating unfavorable conditions for cell division (multiplication). This cytotoxic mechanism is one of the agents responsible for the anticancer effects of DXR (Keizer *et al.*, 1990). The DXR requires a metabolic reduction of the quinone ring for the semiquinone radical to produce a toxic effect (Ramji *et al.*, 2003).

On the other hand, Krishnan and Bastow (2000) also indicated that LAP was involved in interference with the normal function of topoisomerase II (topo II) enzyme. Esteves-Souza *et al.* (2007) showed the inhibitory effects of human DNA-topoisomerase II-a by LAP amine derivatives. According to Esteves-Souza *et al.* (2007) the inhibitory action on DNA-topoisomerase II-a was also evaluated by a relaxation assay of supercoiled DNA plasmid. A significant inhibitory action of the enzyme was observed, and greater activity on the part of LAP amine derivatives than the corresponding lawsone derivatives. Cunha *et al.* (2006) showed that 2 μM of lapachol derivatives significantly hindered topo II-a catalytic activity. This interference in the DNA-Topo II-drug complex may produce a DNA topology that favors the occurrence of recombinational events (Baguley and Ferguson, 1998). Lehmann *et al.* (2003) attributed homologous recombination induced by DXR to a similar mechanism.

Although homologous recombination is an important pathway in DNA repair, there is growing evidence that deleterious genomic rearrangements may result from homologous recombination, which means that homologous recombination events may play a causative role in carcinogenesis (Arossi *et al.*, 2009). The transformation of normal cells into cancer cells is a multistep process, with mitotic recombination as a mechanism involved in bringing about such transformation (Nowell, 1976; Barrett, 1993). In heterozygous cells, bearing a mutant and normal alleles for a tumor suppressor gene, the somatic recombination may turn up to be a promoter of neoplasms by inducing homozygosis of the mutant tumor suppressor, allele (Maher *et al.*, 1993; Sengstag, 1994).

It can be said that LAP, a quinone belonging to the naphthoquinone group, under the experimental conditions mentioned in this study is genotoxically active through recombination, as verified by wing-spot test of *Drosophila melanogaster*. This genotoxicity was only observed in descendants of the crossing of high metabolic bioactivation (HB). These results demonstrate that LAP, in the analyzed concentration, is an indirect genotoxic agent, thus indicating the need for metabolic bioactivation by the cytochrome P450 enzyme.

## Figures and Tables

**Table 1:** Summary of results in the *Drosophila* SMART assay after treatment with Lapachol (LAP). Larvae from Standard (ST) cross and High Bioactivation (HB) cross.

		Spots per fly (Nº of spots); stat. diagnoses^a^								
Series			Small single spots	Large single spots	Twin	Total spots	Spots with *mwh* clone^c^	Mean clone size class	Frequency of clone formation per 10^5^ cells^d^	
DXR (mg/mL)	LAP (μg/mL)	Nº of flies	(1-2 cells)^b^	(> 2 cells)^b^					Observed	Control corrected
ST cross										
*mwh/flr*^*3*^										
0	0	50	0.44 (22)	0.08 (04)	0.00 (00)	0.52 (26)	26	1.88	1.07	
0.125	0	50	0.84 (42) +	0.52 (26) +	0.52 (26) +	1.88 (94) +	68	2.49	2.79	2.86
0	20	60	0.52 (31) -	0.08 (05) i	0.00 (00) i	0.60 (36) -	36	1.83	1.23	0.16
0	40	60	0.32 (19) -	0.07 (04) i	0.02 (01) i	0.40 (24) -	23	2.04	0.79	-0.28
0	60	60	0.35 (21) -	0.10 (06) i	0.03 (02) i	0.48 (29) -	27	2.11	0.92	-0.14
*mwh/TM3*										
0	0	50	0.04 (02)	0.04 (02)		0.08 (04)	4	2.00	0.16	
0.125	0	50	0.10 (05) i	0.06 (03) i		0.16 (08) i	8	2.50	0.33	0.16
HB cross										
*mwh/flr*^*3*^										
0	0	50	0.62 (31)	0.04 (02)	0.04 (02)	0.70 (35)	33	1.64	1.35	
0.125	0	50	2.04 (102) +	0.22 (11) +	0.32 (16) +	2.58 (129) +	113	1.64	4.63	3.28
0	20	60	0.93 (56) +	0.10 (06) i	0.02 (01) i	1.05 (63) +	62	1.55	2.12	0.77
0	40	60	1.08 (65) +	0.13 (08) i	0.07 (04) i	1.28 (77) +	73	1.70	2.49	1.14
0	60	60	1.25 (75) +	0.10 (06) i	0.05 (03) i	1.40 (84) +	81	1.54	2.77	1.41
*mwh/TM3*										
0	0	50	0.44 (22)	0.08 (04)		0.52 (26)	26	1.58	1.07	
0.125	0	50	0.44 (22) -	0.04 (02) i		0.48 (24) -	24	1.46	0.98	-0.08
0	20	50	0.30 (15) -	0.04 (02) i		0.34 (17) -	17	1.29	0.70	-0.37
0	40	50	0.40 (20) -	0.02 (01) -		0.42 (21) -	21	1.48	0.86	-0.20
0	60	50	0.40 (20) -	0.00 (00) -		0.40 (20) -	20	1.20	0.82	-0.25

^a^Statistical diagnoses according to Frei and Würgler (1988): +, positive; -, negative; i, inconclusive; m, multiplication factor. Kastenbaum-Bowman tests, one sided. Probability levels α = β = 0.05. ^b^Including rare *flr*^*3*^ single spots. ^c^Considering *mwh* clones from *mwh* single and twin spots. ^d^Frequency of clone formation: clones/flies/48,800 cells (without size correction). DXR, doxorubicin; LAP, lapachol.
